# Abundance of adverse environmental conditions during critical stages of crop production in Northern Germany

**DOI:** 10.1186/s12302-018-0138-0

**Published:** 2018-04-02

**Authors:** Maximilian Strer, Nikolai Svoboda, Antje Herrmann

**Affiliations:** 1 Institute of Land Use Systems, Leibniz Centre for Agricultural Landscape Research, Eberswalder Straße 84, 15374 Müncheberg, Germany; 2 Research Platform “Data”, Leibniz Centre for Agricultural Landscape Research, Eberswalder Straße 84, 15374 Müncheberg, Germany; 3 Grass and Forage Science/Organic Agriculture, Christian-Albrechts-Universität Kiel, Hermann-Rodewald Str. 9, 24118 Kiel, Germany

**Keywords:** Critical growth stages, Modelling shifts in phenological patterns, Maize, Wheat, Risk of crop production for the North German Plain, Heat and frost stress

## Abstract

**Background:**

Understanding the abundance of adverse environmental conditions e.g. frost, drought, and heat during critical crop growth stages, which are assumed to be altered by climate change, is crucial for an accurate risk assessment for cropping systems. While a lengthening of the vegetation period may be beneficial, higher frequencies of heat or frost events and drought spells are generally regarded as harmful. The objective of the present study was to quantify shifts in maize and wheat phenology and the occurrence of adverse environmental conditions during critical growth stages for four regions located in the North German Plain. First, a statistical analysis of phenological development was conducted based on recent data (1981–2010). Next, these data were used to calibrate the DSSAT-CERES wheat and maize models, which were then used to run three climate projections representing the maximum, intermediate and minimum courses of climate development within the RCP 8.5 continuum during the years 2021–2050. By means of model simulation runs and statistical analysis, the climate data were evaluated for the abundance of adverse environmental conditions during critical development stages, i.e. the stages of early crop development, anthesis, sowing and harvest.

**Results:**

Proxies for adverse environmental conditions included thresholds of low and high temperatures as well as soil moisture. The comparison of the baseline climate and future climate projections showed a significant increase in the abundance of adverse environmental conditions during critical growth stages in the future. The lengthening of the vegetation period in spring did not compensate for the increased abundance of high temperatures, e.g. during anthesis.

**Conclusions:**

The results of this study indicate the need to develop adaptation strategies, such as implementing changes in cropping calendars. An increase in frost risk during early development, however, reveals the limited feasibility of early sowing as a mitigation strategy. In addition, the abundance of low soil water contents that hamper important production processes such as sowing and harvest were found to increase locally.

**Electronic supplementary material:**

The online version of this article (10.1186/s12302-018-0138-0) contains supplementary material, which is available to authorized users.

## Background

The crop yield attained in the field and its variability are both influenced by a range of climate factors, such as radiation, ambient CO_2_ concentration, precipitation, temperature and soil conditions. Variations in environmental conditions from year to year and in response to climate change may result in substantial shifts in the beginning, duration and end of crop developmental stages. Adequate assessment of these shifts by means of crop modelling will promote understanding of the processes affecting the threats to crop production for specific regions and allow the development of adaptation strategies for climate change.

For the North German Plain, agriculturally a highly productive region, climate change is assumed to have a substantial impact on crop production [1, 2]. Shifts in crop phenology, e.g. by a lengthening of the vegetative period due to changes in management or variation of cultivars, exploits more favourable conditions—and has beneficial effects on the yield [3–8]. The extent to which yield will be increased may vary regionally; while the western part of the North German Plain yield may stay at a similar level as that today, the eastern regions might benefit from temperature and radiation changes [7, 9, 10]. In this respect, climate variability is of great importance [11, 12], since 30% of wheat and up to 50% of maize yield variability observed in Western Europe can be attributed to climate variability [13]. Adverse environmental conditions, such as temperature stress, that occur during critical growth stages may result in severe yield loss and negatively affect yield stability [14, 15]. Shifts in adverse environmental conditions are expected for temperate Europe, e.g. heat stress during flowering periods [15, 16] and changes in precipitation distribution [7, 17].

The impact of adverse environmental conditions depends on a crop’s susceptibility in a given growth stage, which is indicated by, e.g. stage-specific temperature thresholds [18, 19]. Consequently, an assessment of shifts in regional phenological development resulting from climate change—as found in various arable crops grown in Germany [3–6]—is fundamental for the assessment of risk to crop yields. Iglesias et al. [20] reported varying risks through shifts in crop phenology for different European regions. Trade-offs stabilising yield variability could also be conceivable, e.g. bringing forward of specific growth stages may reduce the probability of heat stress [12]. Typically, process-based dynamic crop growth models are utilised in assessment studies [20–22]. These models mostly focus on basic crop growth and development processes; however, within the models, they are only capable to focus on a few development-stage-specific responses to environmental stress.

Recent studies have mainly focused on the patterns and impact of adverse environmental conditions [12, 15, 16, 23]. Trnka et al. [15], for instance, performed a general analysis of the abundance of various adverse environmental conditions on European crop production but did not consider critical growth stages. Gobin [16] provided an analysis of shifts of critical growth stages, but the study was restricted to Belgium. For the North German Plain, no study has yet comprehensively analysed the impact of adverse environmental conditions during critical growth stages under the pressure of climate change.

The objective of the current study, therefore, was to identify and evaluate shifts in patterns of adverse environmental conditions during critical growth stages on the North German Plain, as a prerequisite for assessing risks and developing management strategies to improve cropping systems under climate change conditions. The work was conducted within the framework of an interdisciplinary project (https://www.nalama-nt.de [24]), assessing threats of climate change and globalisation and developing a basis for an integrated and sustainable land management for the benefit of the environment and society on the North German Plain.

In the current study, an inventory of the abundance of adverse environmental conditions during critical growth stages was created for wheat and maize grown in four regions representing the North German Plain. The study was based on recent (1981–2010) phenological and weather data. These data furthermore served to calibrate and validate the dynamic crop growth model DSSAT, which then allowed for the assessment of shifts in phenological development and in the abundance of adverse environmental conditions in different climate projections for the period 2021–2050.

## Methods

### Study sites

**Fig. 1:**
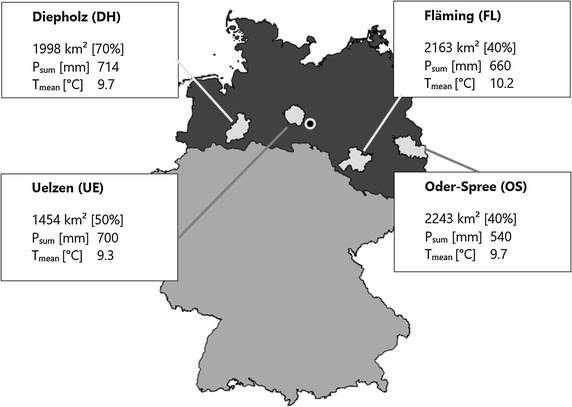
Regions (light grey) located in the North German Plain (dark grey); characterised by total area, cultivated area in percentage of total area (in brackets), average annual precipitation sum (*P*_sum_ [mm]) and annual average temperature (*T*_mean_ [°C]) (Black dot—weather station Salzwedel) [24]

The study area comprised four regions of the North German Plain: Diepholz (DH), Uelzen (UE), Fläming (FL), and Oder-Spree (OS) (Fig. 1). The regions largely correspond to local administration districts—allocated from west to the east along 52°N latitude corridor. The North German Plain is characterised by a temperate oceanic climate (Cfb) in the west and a humid continental climate in the east (Dfb) following the Köppen climate classification [17]. It provides a major fraction of German crop production [24, 25]. In the western regions, fertile silty-loam soils dominate, cultivated with wheat, maize, rapeseed and sugar beet [26]. In the eastern part, shallower sandy to silty-loam soils, are dominant, in which wheat, maize, rye and rapeseed are grown [26]. In the present study, we only considered grain wheat and maize production, common in all regions and of high economic relevance. They represent a winter annual and a summer annual crop, respectively.

### Weather and phenological data

Weather data from representative weather stations in each region were provided at a daily resolution by the German Weather Service (DWD). Phenological data were obtained from DWD database. It comprises sowing dates, the beginning of various phenological stages of wheat and maize in several repetitions for each district in the baseline period (1981–2010) (Fig. 1).

Three climate projections were utilised for future climate evaluation in the projection period from 2021 to 2050 [24, 27]. Ensemble comprised 21 GCM; all were set in the scenario RCP 8.5. For the present study, we selected 3 out of 21 GCM on the basis of their performance in the baseline period and their representation of mean temperature increase in the projection period (2021–2050): a minimum increase of mean temperature to baseline by 1 °C (min, INM-CM4, Russia), an intermediate increase of 1.5 °C (med, ECHAM6, MPI Hamburg, Germany), and a maximum increase of 2 °C (max, ACCESS1.0, CSIRO-BOM, Australia). The utilisation of three different GCM in the RCP 8.5 continuum [27] ensures a wide range of climate change manifestation in respect to e.g. mean temperature or precipitation distribution. Climate data were provided by the Potsdam Institute for Climate Impact Research (PIK). The regionalisation of the GCM output was realised by the statistical analogue resampling scheme (STARS by PIK) at weather station sites.

### Modelling

The decision support system for agro-technological transfer (DSSAT) [22, 28] was used to assess crop phenological development in future climate projections. Calibration for obtaining crop parameter sets was performed on phenological data averaged for the North German Plain (Fig. 1, dark grey, Additional file 1: Suppl Material 1), while validation was based on averaged phenological data within each region (Fig. 1) with weather, soil, and management given as input. The calibration model was set to fit the general environmental conditions of the North German Plain for both crops. The selected phenological time series were prepared by averaging phenological data at various sites throughout Northern Germany for each year to obtain a time series for each phenological growth stage. Weather data for calibration of crop parameter sets was obtained from the centrally located Salzwedel weather station to represent the North German Plain. Soil properties were set to generic medium silty clay (Additional file 1: Suppl Material 2). Such soil types are frequent in fertile alluvial areas throughout Northern Germany (German soil survey (BUEK1000n), [26]). Crop parameters sets were estimated for maize and wheat by minimisation of the root mean square error (RMSE) between simulated and observed phenological data. In addition, goodness of model fit was evaluated in terms of the coefficient of determination (*R*^2^).

**Fig. 2:**
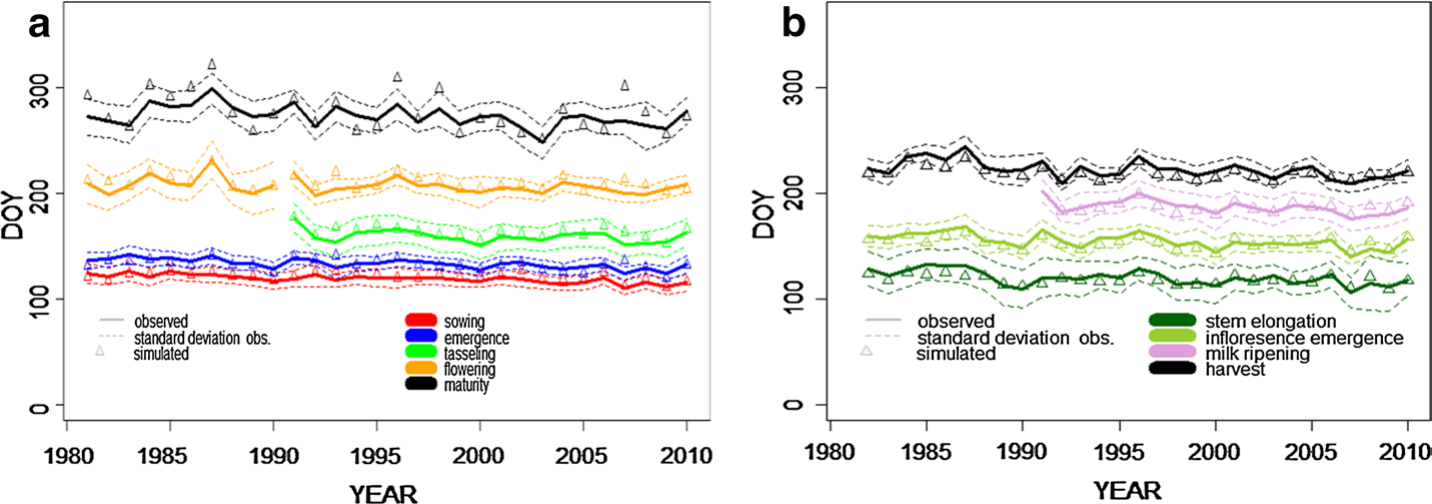
Calibration of phenological development; observed (averaged over the North German Plain) and simulated beginning of specific phenological developmental stages for maize (**a**) and wheat (**b**) on the North German Plain

For validation, crop parameter sets were tested on averaged phenological development time series (DWD) available for each region for the baseline (see Fig. 2, 1981–2010). General production system settings were identical with the calibration procedure. Changes, however, were made to reflect the region-specific environmental conditions, i.e. soils (DH, UE: Additional file 1: Suppl Material 3, FL, OS: Additional file 1: Suppl Material 4, BUEK1000n, [24, 26]), weather conditions (stations of the DWD representative for each of the region, see Fig. 1). Validation was assessed by the coefficient of determination and RMSE for each phenological development stage.

### Data analysis

First, the phenological data were analysed to provide a general description of phenological development for the baseline (1981–2010) and the projection period (2021–2050). For this purpose, linear regression models were fitted to the time series of phenological development with the year as the independent variable and the beginning (day of year) of prominent phenological growth stages of maize and wheat as the dependent variable. The correlated linear model gives information over trends of phenological development in the considered period. Trends were characterised by the slope of the linear regression for each crop in each region. All regression slopes were tested for significance against zero. Statistical analysis was performed utilising GNU R [29]. Generally, significance levels are denoted as follows: “.” for *p* < 0.1, “*” for *p* < 0.05, “**” for *p* < 0.01, and “***” for *p* < 0.001.

Second, the abundance of adverse environmental conditions during critical growth stages of maize and wheat was quantified for the baseline period (1981–2010) and the projection period (2021–2050) in each region. Critical growth stages were defined according to Porter and Gawith [19]; Porter and Semenov [30]; and Sánchez et al. [18] as phenological development stages especially susceptible to adverse environmental conditions. For wheat and maize, the critical stages are provided in Table 1. Adverse environmental conditions were utilised here in the sense of Trnka et al. [15] and Gobin [16] as abiotic environmental events of a relevant length, i.e. days or weeks, which are harmful for crop growth and development. In the present study, we focused on temperature and water limitation, where heat, drought, and frost were analysed on a daily level and heatwaves were analysed for longer periods of time (2 days and more). Furthermore, we included an analysis of high soil water content during sowing and harvest, which is known to be a limiting factor for soil trafficability. Short-term and narrowly localised events exerting mostly rapid physical damage to crops, such as storms, or hailstorms, were excluded from the analysis. The beginning and end of the critical growth stages in question were obtained from DSSAT model runs, and weather data during these stages were evaluated for days exceeding temperature or soil water thresholds as indications of adverse environmental conditions (Table 1). Furthermore, the abundance of drought was evaluated by an assessment of the number of days with soil water content falling below a threshold (Table 1). The percentages of abundance refer to mean growth stage length at each site and each period, respectively, the pre-set number of days evaluated for each crop or around sowing, respectively, maturity in the 30-year period for the for soil moisture.

**Table 1:** Adverse environmental conditions, critical growth stages, and sites especially susceptible to these environmental conditions (*T*_min_—minimum daily temperature, *T*_max_—maximum daily temperature, T_lethal_—lethal temperature for crop development)

Stage	Expected adverse environmental condition	Problem	Sites	Thresholds/limits	References
*Maize*					
Sowing	Soil moisture	Trafficability	Western regions	45% water content (gravimetric)	Trafficability limit 30% water content [29]
Emergence stem elongation	Late frost	Damage organ tissue	Eastern regions	*T*_min_ < 0 °C *T*_lethal_ < − 1.9 °C	[18]
Flowering	Heat	Hampered reproduction	All	*T*_max_ 37.3 °C	[18]
	Heat days		All	*T*_max_ > 30 °C and *T*_min_ > 20 °C	DWD
	Heat spells		All	*T*_max_ > 30 °C following days above limit	DWD
	Drought		All	DH/UE 24% water content FL/OS 12% water content	
Harvest	Soil moisture	Trafficability	DH/UE	45% water content (gravimetric)	Trafficability limit 30% water content [29]
*Wheat*					
Sowing	Soil moisture	Trafficability	Western regions	45% water content (gravimetric)	Trafficability limit 30% water content [29]
Stem elongation–heading	Frost	Damage organ tissue	All	*T*_min_ < 0 °C	[19]
Heading–flowering–milking	Heat	Hampered reproduction	All	*T*_max_ = 31.0 °C following days above limit	[19]
			All	T_max_ > 30 °C and Tmin > 20 °C	DWD
	Drought		All	DH/UE 24% water content FL/OS 12% water content	
Heading–milking	Heat spells	Following days above limit	All	*T*_max_ > 30 °C	DWD
Harvest	Soil moisture	Trafficability	DH/UE	45% water content (gravimetric)	Trafficability limit 30% water content [29]

## Results

### Model performance

Crop parameter sets for maize and wheat were successfully fitted to mean phenological development data (Fig. 2, Additional file 1: Suppl Material 1). Simulated phenological growth stages for maize and wheat mostly lay within the limits of the standard deviation of observed data, e.g. 84% of cases for wheat anthesis and 89% for maize milk ripening (Fig. 2), and the goodness of model fit depended on the phenological development stage. For maize, *R*^2^ values for comparison of simulations and observations over 30 years tended to decrease from sowing to maturity (sowing: *R*^2^ = 0.94 (RMSE = 2.5), emergence: *R*^2^ = 0.83 (RMSE = 3.8), end of juvenile development: *R*^2^ = 0.46 (RMSE = 15.2), flowering: *R*^2^ = 0.54 (RMSE = 9.2), maturity: *R*^2^ = 0.61 (RMSE = 18.7). For wheat, *R*^2^ values remained relatively constant (stem elongation: 0.53 (RMSE = 2.9), inflorescence emergence: 0.59 (RMSE = 3.9), and milk ripening: 0.59, RMSE = 3.8). The onset of maturity, however, was better reflected (*R*^2^: 0.75, RMSE = 3.0).

The model validation revealed comparable results as the model calibration for both crops (Tables 2, 3). Phenological development was predicted reasonably but varied depending on the region, phenological developmental stage and crop, partly due to differences in the amount and quality of data. Restructuring of administration in the course of the German re-unification led to occasionally missing data in the eastern regions. The smallest deviation between observed and predicted values was found for the centrally located UE region. For maize, the developmental stage of tasselling showed an inferior model fit at sites UE and OS (Table 2), while for wheat, simulation of maturity stage was closer to observations than stem elongation, inflorescence emergence and ripening. Simulated maturity at days of the year > 300, which occurred in a few year–region combinations, was due to simulation termination rather than achievement of maturity.

**Table 2:** Model validation for the beginning of different phenological developmental stages of maize, specified as day of year

	Sowing		Emergence		Tasselling		Flowering		Maturity	
	*R* ^2^	RMSE	*R* ^2^	RMSE	*R* ^2^	RMSE	*R* ^2^	RMSE	*R* ^2^	RMSE
DH	0.80	2.54	0.80	3.02	0.40	13.96	0.57	6.08	0.90	14.86
UE	1.00	0.25	0.56	3.42	0.17	17.78	0.38	8.43	0.87	21.95
FL	0.80	2.31	0.39	5.18	0.46	7.18	0.60	4.14	0.61	11.96
OS	1.00	0.29	0.57	5.84	0.18	10.92	0.84	11.77	0.45	17.37

Goodness of model fit is provided as the coefficient of determination (*R*^2^) and root mean square error (RMSE)

**Table 3:** Model validation for different phenological developmental stages of wheat, specified as day of year

	Stem elongation		Inflorescence emergence		Ripening		Maturity	
	*R* ^2^	RMSE	*R* ^2^	RMSE	*R* ^2^	RMSE	*R* ^2^	RMSE
DH	0.36	7.06	0.58	4.11	0.36	9.07	0.68	7.11
UE	0.6	4.48	0.63	4.13	0.63	5.02	0.73	6.13
FL	0.53	5.18	0.53	4.85	0.64	7.81	0.90	12.27
OS	0.21	7.46	0.47	5.95	0.07	14.9	0.66	3.76

Goodness of fit is provided as the coefficient of determination (*R*^2^) and root mean square error (RMSE)

The deviation between observed and modelled dates in the sowing of maize indicated by a relatively low *R*^2^ (0.8) in DH and FL is due to the comparison of sowing dates as the means from observed, regional data (Table 2) and the actual, natural numbered input data for the simulation.

### Shifts in phenology in the recent data set

**Fig. 3:**
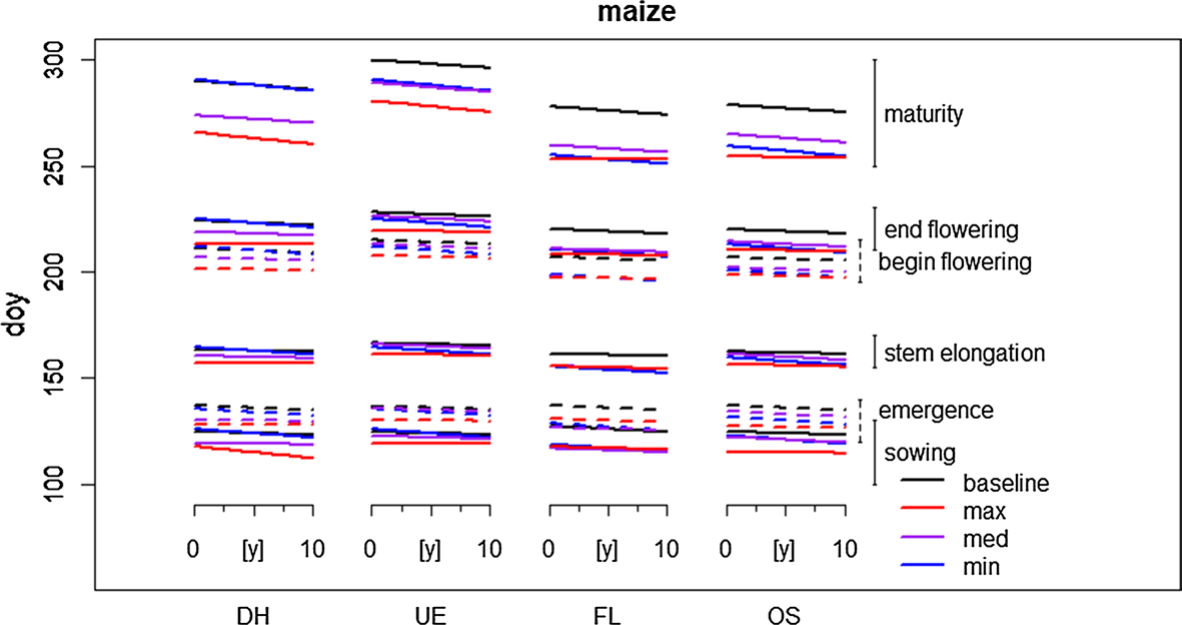
Changes in maize phenological development during the projection and baseline periods as linear trends in the four regions (dashed/solid lines and brackets for differentiation of overlapping clusters of phenological stages; see also Table 6, and Additional file 2: Suppl Materials 5–10)

The observed phenological data showed shifts to earliness for various phenological stages of both crops. In maize, tendencies towards earlier occurrence—indicated by the slopes of linear regression models—were identified for nearly all developmental stages (Fig. 3, Table 4). An exception was emergence in OS (0.09 ± 0.13 d/y or 1.2 ± 1.7 d/°C), where *R*^2^, i.e. the portion of the phenological time series development described by the linear trend was very low (< 0.01), as well as tasselling in UE (0.27 ± 0.18 d/y or 3.47/± 2.25 d/°C, *R*^2^ < 0.01) and in DH (0.09 ± 0.17 d/y or 1.3 ± 2.15 d/°C, *R*^2^ < 0.01). Generally, the number of significant trends identified was higher in DH and UE, i.e. three out of five trends. In contrast, in the OS region, only one out of five trends was significant (Table 4). This might be attributed to smaller sample sizes caused by less observation sites in these areas and a more fragmentary data structure.

**Table 4:** Linear regression parameters quantifying the changes in maize phenological development for the observed phenological data of the four regions during the baseline period

	DH			UE			FL			OS		
	Estimate	*R*^2^/*n*	*p* value	Estimate	*R*^2^/*n*	*p* value	Estimate	*R*^2^/*n*	*p* value	Estimate	*R*^2^/*n*	*p* value
	[d] [d/y]			[d] [d/y]			[d] [d/y]			[d] [d/y]		
Sowing	119 ± 7	0.14		122 ± 7	0.02		118 ± 7	0.1		120 ± 7	0.01	
	− 0.35 ± 0.05	258	4.3E−10***	− 0.14 ± 0.07	187	5.1E−02	− 0.46 ± 0.20	55	2.2E−02*	− 0.1 ± 0.21		6.3E−01
Emergence	134 ± 8	0.17		134 ± 8	0.06		131 ± 7	0.12		133 ± 6	0	
	− 0.4 ± 0.06	255	9.1E−12***	− 0.24 ± 0.08	177	2.0E−03**	− 0.52 ± 0.19	56	9.0E−03**	0.06 ± 0.18		7.4E−01
Tasselling	192 ± 12	0		194 ± 15	0.02	1.4E−01	202 ± 23	0.16		198 ± 15	0.16	
	0.09 ± 0.13	168	4.8E−01	0.27 ± 0.18	125		− 1.21 ± 0.31	81	0.0E+00***	− 0.84 ± 0.25		1.0E−03**
Flowering	201 ± 10	0.05		206 ± 9	0.06	1.6E−02*	201 ± 7	0		199 ± 17	0.01	
	− 0.36 ± 0.16	108	2.6E−02*	− 0.45 ± 0.18	90		− 0.06 ± 0.22	50	7.9E−01	− 0.28 ± 0.59		6.4E−01
Harvest	280 ± 17	0		271 ± 18	0.01	1.7E−01	262 ± 15	0.08		262 ± 11	0.03	
	− 0.07 ± 0.13	259	5.8E−01	− 0.24 ± 0.17	196		− 0.8 ± 0.39	54	4.4E−02*	− 0.4 ± 0.34		2.5E−01

Significance levels are denoted as followed: “.” for 0.1, “*” for 0.05, “**” for 0.01, and “***” for 0.001

For wheat, phenological development shifted forward several days at all sites. The linear trends, however, were not always significant, which, as seen in maize, is probably due to the availability and quality of phenological data. For instance, in DH and UE, eight out of the twelve significant trends had three times larger sample sizes than corresponding data sets for the eastern sites. Slopes derived for the eastern regions, however, were comparable to those obtained for western regions. The period around anthesis, i.e. the most critical growth stage, became shorter, as indicated by trends for inflorescence emergence of 0.28 ± 0.99 d/y (OS, respectively, 3.7 ± 13.4 d/°C) and − 0.23 ± 0.20 d/y (FL, respectively, − 2.3 ± 2.0 d/°C) and for milk ripeness of − 0.84 ± 1.22 d/y (OS, respectively − ,11.4 ± 16.5 d/°C) and − 2.01 ± 0.725 d/year (FL, respectively, − 20.1 ± 7.25 d/°C), respectively (Table 5).

**Table 5:** Linear regression parameters quantifying the changes in wheat phenological development in the four regions during the baseline period

	DH			UE			FL			OS		
	Estimate	*R*^2^/*n*	*p* value	Estimate	*R*^2^/*n*	*p* value	Estimate	*R*^2^/*n*	*p* value	Estimate	*R*^2^/*n*	*p* value
	[d] [d/y]			[d] [d/y]			[d] [d/y]			[d] [d/y]		
Stem elongation	118 ± 13	0.09		123 ± 16	0.01		126 ± 9	0.07		129 ± 42	0.02	
	− 0.48 ± 0.12	162	0.0E+00***	− 0.23 ± 0.14	202	9.3E−02	− 0.52 ± 0.41	25	2.2E−01	− 1.26 ± 2.19	18	5.7E−01
Flowering	155 ± 9	0.13		156 ± 11	0.13		155 ± 12	0.02		157 ± 33	0	
	− 0.41 ± 0.08	169	1.3E−06***	− 0.49 ± 0.09	218	4.2E−08***	− 0.23 ± 0.20	56	2.6E−01	0.28 ± 0.99	22	7.8E−01
Milk ripeness	191 ± 14	0.35		187 ± 13	0.05		187 ± 14	0.34		172 ± 20	0.05	
	− 1.34 ± 0.22	69	9.1E−08***	− 0.52 ± 0.23	104	2.2E−02*	− 2.01 ± 0.73	17	1.4E−02	− 0.84 ± 1.22	12	5.1E−01
Harvest	224 ± 12	0.2		224 ± 12	0.11		221 ± 14	0.01		214 ± 11	0	
	− 0.66 ± 0.10	180	4.0E−10***	− 0.51 ± 0.10	224	4.2E−07***	0.32 ± 0.63	25	6.2E−01	0.07 ± 0.38	30	8.5E−01
Sowing	289 ± 16	0		287 ± 12	0.08		285 ± 9	0.05		286 ± 18	0	
	0.08 ± 0.16	175	5.8E−01	− 0.43 ± 0.10	224	2.8E−05***	− 0.43 ± 0.42	23	3.2E−01	− 0.23 ± 0.64	31	7.3E−01
Emergence	299 ± 33	0.01		301 ± 15	0.08		301 ± 10	0.03		299 ± 19	0	
	− 0.44 ± 0.32	164	1.8E−01	− 0.54 ± 0.13	210	3.2E−05***	− 0.39 ± 0.46	23	4.1E−01	− 0.1 ± 0.68	30	8.8E−01

Significance levels are denoted as followed: “.” for 0.1, “*” for 0.05, “**” for 0.01, and “***” for 0.001

### Shifts in phenology in the projection period

**Fig. 4:**
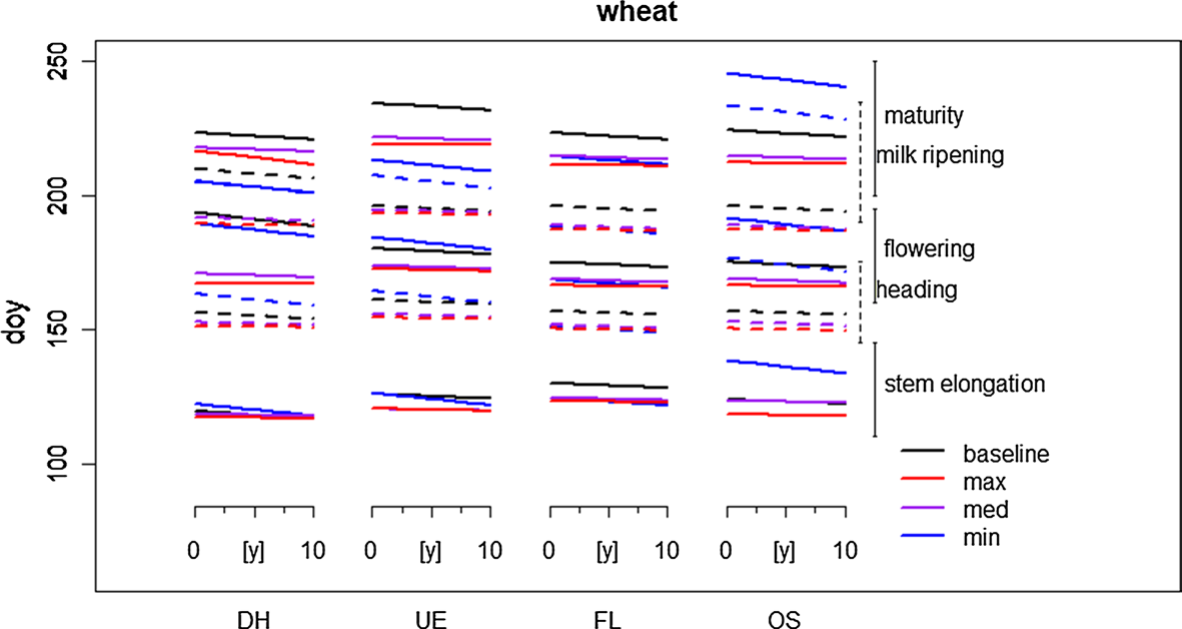
Changes in wheat phenological development during the projection and baseline periods as linear trends in the four regions (dashed/solid lines and brackets for differentiation of overlapping clusters of phenological stages; see also Table 7, and Additional file 2: Suppl Materials 5–10)

The shifts in phenology found for the future climate projections are presented in detail for region DH (Additional file 2: Suppl Materials 5 and 9, Figs. 3, 4). The response patterns quantified for the remaining regions were similar and were strongly correlated to the temperature increase of the projections, i.e. growth stages show similar behaviour for the temperature levels in the projection period in each region (Additional file 2: Suppl Materials 5–12). Phenological development in the DSSAT-CERES model is influenced by temperature. Consequently, critical growth stages of maize and wheat occurred earlier, and the duration shortened in the projection period. Shifts were consistent with those identified in the baseline period.

For maize, a forward shift of several days was found for sowing and each consecutive growth stage in all projections at all locations (Fig. 3). A tendency was found for the acceleration to be larger in later growth stages because the temperature effect is cumulative, and the maximum projection which was to chosen as to show the highest temperature increases generally showed the strongest effects compared to the baseline period. Duration and earliness of anthesis were clearly correlated with the mean temperature increase in each of the three projections (Additional file 2: Suppl Material 5, Fig. 3). For maturity, earliness adds up to more than 2 weeks for the max projection (Additional file 2; Suppl Material 5, Fig. 3). The determination of maize harvest, with respect to maturity stage, was generally accompanied by larger uncertainties.

For wheat, a forward shift of phenological stages was also found for all regions (Additional file 2: Suppl Material 9, Fig. 4). As expected, this response was correlated to the increase in mean temperature in the projections. In intermediate and minimum, the shift was only a few days in the maximum projection maturity occurred up to 2 weeks earlier compared to the baseline (Additional file 2: Suppl Material 9, Fig. 4). Like maize, the forward shift was most pronounced for maturity. The length of the critical growth stage around flowering was reduced by 1 day, with the maximum projection showing the largest effect (Additional file 2: Suppl Material 9). Only UE deviated from this pattern, where we found an increase of 3 days for the projected rather than a decrease (Fig. 4). In addition, the interval of stem elongation to inflorescence emergence in wheat increased by approximately 6 days in the projection period. An explanation is that photoperiod hampers accumulation of degree days that propels phenological development. Thus, despite increased mean temperatures, phenological growth stages are elongated.

### Adverse environmental conditions

The abundance of adverse environmental conditions increased during critical growth stages in the future projections (Tables 6, 7). All regions showed similar general behaviour in the earliness of phenological development and shifts in the abundance of various adverse environmental events (Tables 6, 7). However, some specific features, e.g. soil moisture and number of hot days, indicate differences between west and east.

**Table 6:** Abundance of adverse environmental conditions (fraction, number of days) during specific development stages of maize denoted by BBCH stadium [31] in the four regions for the baseline (base, 1981–2010) and projected projections (max, med, min; 2021–2050) and the abundance of heat spells with certain lengths (indicators as given in Table 1)

Stage	DH				UE					FL				OS		
BBCH	Base	Max	Med	Min	Base	Max	Med	Min	Base	Max	Med	Min	Base	Max	Med	Min
01–30																
*T*_min_ < 0 °C	0.2	1.2	1.3	1.3	0.5	2.5	1.4	1.9	0.2	1.4	1.7	1.3	0.3	0.9	0.9	1.4
*T*_min_ < 10 °C	68.0	63.0	60.0	64.0	66.0	64.0	61.0	65.0	60.0	62.0	61.0	60.0	55.0	56.0	54.0	56.0
øn [d]	42.7	43.4	41.0	41.6	44.0	42.9	42.5	42.3	40.8	40.8	41.4	40.4	39.7	40.4	40.5	40.7
31–60																
*T*_min_ < 0 °C	0.0	0.0	0.0	0.0	0.0	0.1	0.1	0.1	0.0	0.0	0.0	0.0	0.0	0.0	0.0	0.0
*T*_min_ < 10 °C	18.0	18.0	20.0	16.0	19.0	22.0	22.0	19.0	16.0	17.0	17.0	16.0	13.0	11.0	12.0	11.0
øn [d]	46.1	46.1	46.3	45.2	48.1	46.3	48.3	46.7	44.4	43.1	44.0	44.2	45.0	42.1	43.4	43.6
61–70																
*T*_max_ > 37.3 °C	0	0	0	0	0	0	0	0	0	0	0	0	0	0	0	0
Heat	0.3	0	0	0.3	0	0	0	0	1.1	0.3	0.3	0.3	2.6	1.8	1.2	1.5
Drought	5.8	14.7	15.5	9.4	5.9	9.6	9.3	8.2	7.8	3.4	5.6	5.3	2.1	3.4	4.8	6.3
øn [d]	12.3	13.1	12.4	13.2	12.6	12.9	12.7	13.9	12.1	11.5	12.1	11.9	11.8	11.4	11.8	11.9
70–99																
*T*_max_ > 36 °C	0.2	0.6	0.3	0.1	0.1	0.1	0.1	0.0	0.5	1.2	0.5	0.6	0.3	1.1	0.2	0.0
Heat	0.1	0.2	0.1	0.1	0.0	0.1	0.0	0.0	0.5	1.2	0.5	0.2	0.8	2.1	1.4	0.7
øn [d]	60.5	50.6	49.5	53.6	66.4	53.4	56.4	62.2	52.5	41.5	44.5	47.4	54.9	42.2	45.3	47.1
61–70																
Heat spell 1	15	16	15	13	12	13	17	12	20	22	19	14	12	15	17	18
Length [d] 2	4	4	11	4	4	5	3	3	5	7	8	12	8	9	12	7
3	3	3	1	3	2	3	1	1	3	5	3	3	–	2	1	3
4	2	3	–	–	1	2	1	1	–	1	1	2	–	2	1	2
5	2	1	–	–	1	–	1	–	–	–	1	2	1	–	1	–
6	–	–	–	1	1	–	1	–	1	1	1	–	1	–	1	–
7	1	1	–	–	–	–	–	–	–	–	–	1	1	–	–	.

**Table 7:** Abundance of adverse environmental conditions (fraction, number of days) during specific development stages of wheat denoted by BBCH stadium [31] in the four regions for the baseline (base, 1981–2010) and projected projections (max, med, min; 2021–2050) and the abundance of heat spells with certain lengths (indicators as given in Table 1)

Stage	DH				UE				PM					OS		
BBCH	Base	Max	Med	Min	Base	Max	Med	Min	Base	Max	Med	Min	Base	Max	Med	Min
31–50																
*T*_min_ < 0 °C	0.3	0.7	1.5	1.1	0.6	1.5	1.5	1.0	0.0	0.3	0.5	0.3	0.2	0.3	0.3	0.9
øn [d]	21.9	33.3	33.4	33.5	22.0	33.5	33.9	34.0	17.2	26.7	26.7	26.8	20.8	31.5	27.2	31.6
51–60																
*T*_max_ > 25 °C	21.0	28.0	30.0	27.0	16.0	23.0	24.0	20.0	25.0	39.0	35.0	31.0	22.0	36.0	33.0	27.0
*T*_max_ > 31 °C	1.5	3.1	3.9	3.5	0.6	1.6	2.2	1.6	2.6	3.2	5.7	6.2	1.8	3.5	4.0	4.4
øn [d]	18.0	17.5	16.9	16.7	18.1	17.5	17.2	17.3	17.3	16.0	16.2	16.1	17.1	15.9	16.3	16.4
51–75																
*T*_max_ > 31 °C	3.9	4.6	4.6	4.0	1.3	3.2	2.6	2.2	5.9	6.3	6.7	5.6	4.2	4.9	5.6	4.3
Heat DWD	0.3	0.2	0.0	0.0	0.0	0.0	0.0	0.0	0.2	0.2	0.1	0.1	0.7	0.7	0.5	0.1
Drought	14.5	24.7	13.1	14.3	14.4	27.9	21.9	13.9	12.2	12.4	14.8	12.5	10.7	11.7	10.1	12.1
øn [d]	39.0	37.9	38.2	38.0	34.2	38.1	39.1	39.0	37.4	35.8	36.4	36.6	37.3	35.4	36.2	36.6
51–75																
Heat spell 1	17	24	24	25	5	17	12	12	20	20	26	29	17	24	28	23
Length [d] 2	5	3	4	5	2	7	3	2	12	8	10	1	7	5	10	7
3	2	5	5	3	1	–	1	2	3	3	6	–	3	2	1	–
4	–	–	1	–	–	1	1	1	1	2	1	1	1	1	1	2
5	1	1	–	–	–	–	–	–	–	1	–	–	–	1	–	–
6	–	–	–	–	–	–	–	–	–	1	–	–	–	–	–	–

#### High temperature

Generally, climate change projections with larger temperature increases caused a greater abundance of high-temperature events, whereas the length of critical growth stages for maize and wheat decreased (Tables 6, 7). The occurrence of high temperatures during maize anthesis and in the post-anthesis phase, however, was rare. In particular, daily maximum temperature exceeding 37 °C [18] did not occur around anthesis, neither in the baseline period nor in the projections (Table 6). Only several days into the post-anthesis phase the temperature exceed 36 °C (data not shown). Similarly, only very few hot days, i.e. days with *T*_max_ > 30 °C and *T*_min_ > 20 °C were detected around anthesis for the baseline period. For the projections, an increase in high-temperature events was found, which correlated with the projections’ mean temperatures (Table 7). For instance, the abundance of hot days in the post-flowering phase of maize (BBCH 71-99) increased from 0.06% in the minimum projection to over 0.14% in the intermediate projection to 0.2% in the maximum projection for DH. In addition, hot days during anthesis were rare in the western regions, DH and UE, with only a few days in the baseline and minimum projection in DH, whereas in eastern regions, there were 10 hot days recorded in the baseline period. Moreover, this period was shortened by approximately 1 day.

For wheat, an increase in the exceedance of almost all investigated temperature thresholds was found during the critical growth stage between flowering and milk ripeness (Table 7), with the risk increasing with mean temperature increase in the projections. The number of heat spells in the interval between inflorescence emergence and milk ripeness increased from the baseline to the projection period throughout all sites and for all heat spell lengths. In addition, for FL, heat spells > 6 days were detected, which had not yet been recorded (Table 7).

#### Low temperature

Temperatures below the base temperature for maize (10 °C) occurred with similar or lower frequency between sowing and tasselling in the climate projections (Table 6). Temperatures below 0 °C between sowing and inflorescence emergence were rare in the baseline. For instance, we found three underruns in DH in the baseline period and approximately 15 in the projections (Table 6). Underruns of the minimum temperature thresholds never occurred in the interval between stem elongation and tasselling at any site (Table 6). In the projections, some isolated frost days (1 or 2 each) only occurred at the UE site.

Similar results were found for frost during the early development of wheat. In the baseline, frost was rare or non-existent between stem elongation and inflorescence emergence for all regions (Table 7). In the climate projections, frost occurred approximately 5 times more frequently for wheat. Two days, for instance, were found in the baseline period compared to a range of 7–15 days in the projections (Table 7). While a clear difference was found between the baseline and projection periods, the extent was arbitrary among the projections, where no direct relation between projection temperature and number of frost days was detected. Obviously, higher probabilities for extreme temperature are promoted despite beneficial shifts in mean temperature. This contrasts with the high temperature threshold exceedances and heat days, where mean projection temperature increase was correlated to the abundance of high-temperature events.

#### Soil water

For the analysis of soil hydrological conditions, the exceedance of modelled soil water content (> 45% in top soil to a depth of 30 cm) was evaluated for each projection and each site (Table 8). The analysis was set to a period of ± 5 day around sowing date for each year separately as well as 10 days around harvest, which was provided by the model as maturity date. Soil water content never limited trafficability in the FL and OS regions (data not shown).

**Table 8:** Soil water conditions (abundance of days with water content, *θ*, over 0.45 in the top 30 cm of soil, *n*—gives the number of days evaluated for each crop on or around sowing, respectively, maturity for the 30-year period) predicted for sowing and harvest in DH and UE

Crop	Stage		DH				UE			
	[BBCH]	*n*	Base	Max	Med	Min	Base	Max	Med	Min
Maize	01	30	0	11	27	29	1	18	25	29
	01 ± 5 d	300	0	107	302	310	12	182	273	309
	99	30	25	22	22	26	24	24	25	28
	99 − 9 d	300	247	221	216	265	252	236	233	269
Wheat	01	29	18	12	17	19	21	13	14	20
	01 ± 5 d	290	194	139	117	207	218	161	127	232
	99	29	11	4	11	17	12	4	13	8
	99 − 9 d	290	57	15	56	164	63	22	70	33

For maize, high soil water contents at sowing rarely occurred in the baseline, whereas in the projections, the number of days with soil water content > 45% increased up to 29 in DH as well as in UE. Furthermore, a clear gradation became apparent among the projections, with the maximum projection leading to the smallest number, and the minimum projection leading to the largest number of days with high soil water content. This differs from the pattern found for maturity, where the baseline and projections were generally equivalent. The abundance of the actual date and the time span around that date were similar for sowing and maturity.

For wheat, days with high soil water content at sowing were similar for both sites, i.e. approximately 60% out of the 29 years in the baseline (Table 8), while for maturity only approximately 30% of days were above the threshold. The projections revealed generally the same pattern as for maize, with the maximum projection having the lowest abundance and the minimum projection showing the most days above the threshold. The baseline was similar to the minimum and intermediate projections.

The evaluation of low water content as an indicator of drought at the four regions (Tables 6, 7) shows high variability between baseline and projections for maize and wheat (Table 1). The western regions showed an increase of percentage of days below the soil water threshold during flowering in the med and max projections, in particular at sites DH and FL. The eastern regions revealed an opposite trend. This was partly due to single severe drought events as the year 2003 which had strong impact on the abundances identified. The comparison of wheat and maize revealed a more pronounced increase of drought conditions for wheat, in particular between inflorescence emergence and milk ripe.

## Discussion

### Phenology

The shift in the phenological development documented for maize and wheat in the baseline period is in accordance with various studies conducted for Germany and Europe [3–5, 32, 33]. Menzel et al. [5], for instance, reported a 2.5 days/°C earlier occurrence of phenological stages in the spring, translating to 2.5 days per decade for various crops grown in Europe. Similarly, an earlier phenological development of 2–2.9 days per decade was found when analysing statistical data from 1960 to 2000 for Germany [3]. For wheat, e.g. the beginning of inflorescence emergence was found to advance by 2 days per decade in Germany, which is considerably less than our finding of 3–5 days per decade (Table 5). For maize, full flowering on average was found to shift forward by 0.47 days per decade [4] in Central Europe, which is in good agreement with our study, where a shift of 2–3 days earlier was documented over 30 years (Table 4). Comparability among studies is limited due to differences in the evaluated time spans, phenological data availability and regional context. Warming patterns are regarded as the main cause for phenological shifts [5]. Other factors influencing crop development, however, cannot be disregarded, such as management [3, 4] or shifts in cultivars.

The lack of significance in some of the identified trends, especially during the baseline period of the OS and FL regions can be attributed to discontinuous time series and small sample sizes. The lack of significance in the trend for the sowing date of wheat in DH probably is due to limited machinability in late summer/early fall caused by water-saturated soils [16]. Furthermore, labour shortages can lead to rigid schemes for sowing. This is the case especially for smaller farm sizes [25].

The shifts in phenological development identified for the projection periods in the current study are comparable to those reported by other studies for European conditions [15, 34]. Schröder et al. [34], for instance, found an advancement of up to 10 days of phenological stages in the first half year, based on simulations by 10 climate models for the period 2031–60 (temperature projection +3.7 °C in 2100) for Hessen, Germany.

### Model performance

Phenological development was reasonably well predicted for all regions of the North German Plain. Deviations between simulated and measured values were mostly within the standard deviation (Fig. 2, Tables 2, 3). This is in agreement with Palosuo et al. [21], who found DSSAT to be capable of reproducing the anthesis (EC 61) and yellow ripeness (EC 90) dates of European wheat production, with comparable RMSE of approximately 6 days for anthesis and 8 days for yellow ripeness. For maize, Vučetić [33] found satisfying results in predicting the phenology of maize in Zagreb, Croatia, predicting silking with *R*^2^ = 0.71 and maturity with *R*^2^ = 0.66, which is within the range documented in the present study (Table 2). Somewhat larger discrepancies became evident for the maize harvest, as indicated by high standard errors of up to 3 weeks (Tables 2, 3). Most likely this is due to the underlying database, where harvest was not differentiated among different production types, i.e. silage maize, corn cob mix and grain maize. The harvest date provided by DSSAT maize is physiological maturity, but the phenological data recorded in the North German Plain will contain a considerable proportion of maize harvested at silage maturity. In this respect, the different maturation behaviour of silage maize with respect to the maturity group and the maturation of stover compared to cob may have further contributed to larger deviations between the observed and simulated data. Nevertheless, the calibration parameter set can be regarded as valid to describe the phenological development of maize and wheat in the four regions. This is particularly true since other environmental factors, such as local water and nutrition supply, are generally not considered for phenological development in crop models [28].

### Adverse environmental conditions

Thresholds are commonly used in crop models as indicators of adverse environmental conditions. Physiological stress, however, is not a result of threshold exceedances, but a complex interaction of the environmental history of a site finally leading to effects on plant growth processes. In this respect, interactions of abiotic stress factors [35] or acclimatisation effects [30] may substantially vary the extent of the environmental impact on growth and development processes. It has also been shown that abiotic state variables are not necessarily highly correlated with plant-response mechanisms [36]. Thresholds, however, are easily accessible, and the difference between abundances in the baseline period and the projections is a suitable indicator for changes in environmental patterns [15, 16].

**Fig. 5:**
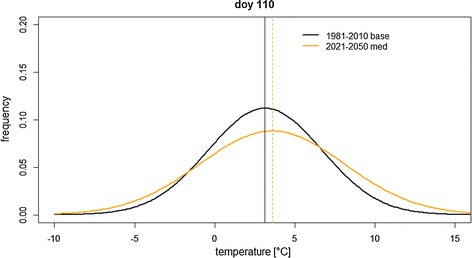
Example of normal distributions fitted to detrended low temperatures at the DH site for the baseline 1981–2010 and the projection period at doy 110

The increased abundance of environmental conditions exceeding thresholds in the current work is similar to other studies reporting an increase of heat and drought stress all over Europe [15, 16]. For maize, however, heat stress around anthesis seems less relevant in the North German Plain, since the threshold value was not exceeded in either the baseline or the projection periods. Although there was an increase in hot day events in the projection period, these days were still beneath the anthesis lethal temperature threshold of 37 °C [18]. It should also be considered that despite increased mean temperatures, we found shifts in the distribution of temperatures that would increase the probability of low-temperature abundance (Fig. 5). With respect to low temperatures (< 0 °C), the current study documented a fourfold higher abundance of frost occurrences in the projections compared to the baseline for the period from sowing until stem elongation in maize. The same pattern was found for wheat.

While low temperatures can have a significant impact on the development of maize, the impact of low temperature per se should be less pronounced for wheat [18, 19]. The increased abundance of lower temperatures can be explained through the earlier phenological development in both crops. While increased mean temperatures promotes an earlier phenological development in the crop model, shifts in temperature distributions in the projected climate can increase the abundance of lower temperature (Fig. 5). In addition, photoperiod and frost effects in the crop model hamper the accumulation of degree days and lengthen specific phenological stages, especially in winter wheat production, which is completely exposed to the period with short day length in Northern Germany. This suggests that frost conditions can be a reasonable threat in future German cropping systems. Similarly, Trnka et al. [15] identified an increased abundance of late frost for wheat production systems at several investigated sites, and increased winter frost abundance at continental sites in Europe. In contrast, Gobin [16] reported maize and wheat to benefit from earlier planting in Belgium. However, late frost abundance was not investigated in that study.

An evaluation of the impact of adverse environmental conditions on crop growth and development, whether it be for historical or future periods, is always afflicted by uncertainty, since adverse environmental conditions are rare events and thus a general source of error [23]. Despite the use of 30-year time slices, small case numbers inhibited further statistical analysis for significance, and the analysis therefore was only descriptive. However, temperature-related effects were consistent.

Soil trafficability during sowing and harvest can be a limiting factor in crop production, but it strongly depends on local soil properties. In the current study, shifts in soil water conditions were small and arbitrary. We were not able to identify clear trends between the baseline and projection time periods for most sites. If a change occurred, it was an increase; however, changes were inconsistent over the three evaluated projections. This is in contrast to Gobin [16], who found that the number of water-logged days at the time of planting for summer crops as well as for the harvesting of maize declined from 1947 to 2008 in Belgium.

Our analysis of drought abundance during critical development stages of maize—indicated here by days with soil water content falling below a threshold—gives only an overview on the complexity of precipitation distribution in a climate change context. The abundance of drought events was correlated to single severe drought events as in the year 2003 [39], which had strong impact on the abundances identified. The utilisation of accumulative methods quantifying drought, or other standardised indicators including precipitation and evapotranspiration [16, 37] would clearly improve the assessment of drought in itself. The model, however, is not yet validated for evapotranspiration. In addition, the impact of carbon dioxide concentration on crop transpiration is not included in the model [38]. However, some features are reasonably explainable. Wheat drought abundance in FL and OS is in accordance to the climate change scenarios, where higher annual precipitation together with a shift to more winter rainfall resulted in nearly constant summer precipitation [27] (Table 9). The reduced drought abundance detected in maize can be attributed to typical heavy rain events in the summer replenishing soil water [7, 17], especially in the more continental Eastern regions.

**Table 9:** Mean annual precipitation sums for the baseline and the projection periods at the for regions (percentage gives ratio of precipitation for the April–September period)

Region	Baseline (mm)	Min (mm)	Med (mm)	Max (mm)
DH	705 (52%)	739 (0.48)	746 (47%)	705 (48%)
UE	732 (53%)	758 (50%)	742 (48%)	728 (48%)
FL	542 (56%)	578 (50%)	560 (48%)	545 (47%)
OS	551 (59%)	576 (51%)	562 (49%)	564 (47%)

Temperature shifts can be explained consequently throughout the regions by the mean temperature increase given by climate scenarios [40]. The STAR scheme has proven to be reliable to break down general circulation models (GCM) to regional levels [41]. However, precipitation provided in the climate models is regional and within each projections highly variable. GCM shows a higher variability in predicting hydrological aspects than in predicting temperature [42, 43]. Similarly, Ljungqvist et al. [43] emphasised that precipitation as provided by GCM is highly variable and should be considered as random manifestation rather than being interpreted in a context of expectable shifts.

## Conclusions

The increased abundance of temperature-related stress in all projections indicates the necessity of improving cropping systems to minimise the risk for crop production in the North German Plain. This particularly applies to the eastern North German Plain, where a stronger impact of climate change may be expected, and requires the development of adaption strategies. Apart from breeding for more stress-tolerant genotypes—primarily heat tolerance around anthesis in wheat and cold tolerance for germination and early development in maize—there is potential for earlier sowing of summer-annual cultivars to avoid high temperatures and drought during critical development stages, i.e. flowering in early summer. For maize, earlier sowing, however, could result in a trade-off due to the risk of frost damage. For winter annuals, such as wheat, earlier maturing genotypes might be an option to ensure that reproductive development will occur under more favourable environmental conditions. Changes in soil water content affecting trafficability were small but should not be ignored.

The methodological approach applied in the current study is easily transferable to other adverse environmental conditions, e.g. by selecting indicators of moisture-limitation. A methodological challenge exists because of small sample sizes, which are a consequence of the moderate climate in the region, and, in the case of critical development stages, of the fine-tuned and specifically adapted production systems. Another challenge lies in the crop models’ capabilities of predicting phenological growth stages. In general, crop growth models have proven to be suitable for predicting the phenological development of various crops for this region. However, predicting phenological development under stress conditions, e.g. heat, drought, and multiple stresses, is still a challenge in crop modelling. A refined implementation of stress reactions in crop growth models, i.e. water and heat stress, would allow for a more reliable assessment. For the input site, the quality of the global circulation models is crucial, particularly the aspects related to precipitation.

The method applied in the current study is easy transferable to other regions—provided an adequate set of climatological and phenological data and a suitable crop growth model are available- and gives a reasonable overview on local cropping systems and the abundance of adverse environmental conditions as an indicator for risk assessment.

## Additional files

**Additional file 1.** Suppl Materials 1–4.

**Additional file 2.** Suppl Materials 5–12.
