# Supplementary material for: Abundance of adverse environmental conditions during critical stages of crop production in Northern Germany
Source: Environ Sci Eur. 2018 Apr 2;30(1):10. doi: 10.1186/s12302-018-0138-0 (PMC5880854; doi:10.1186/s12302-018-0138-0)
Supplement: Supplementary file 1 — Additional file 1. Suppl Materials 1–4. [file 12302_2018_138_MOESM1_ESM.docx]

# Additional file 1

Supplementary Material 1 DSSAT model parameters controlling the phenological development of wheat and maize.

| Name | Definition | Value |
| --- | --- | --- |
| **Maize** |  |  |
| P1 | Degree days (base 8°C) from emergence to end of juvenile phase | 220 |
| P2 | Photoperiod sensitivity coefficient (0 -1) | 0.3 |
| P5 | Degree days (base 8°C) from silking to physiological maturity | 730 |
| G2 | Potential kernel number | 670 |
| G5 | Potential kernel growth rate (mg/(kernel d)) | 8.5 |
| PHINT | Thermal time between the appearance of leaf tips (8Cd) | 38.9 |
| **Wheat** |  |  |
| P1D | Photoperiod sensitivity coefficient (% reduction/h near threshold) | 50 |
| P1V | Vernalisation sensitivity coefficient (%/d of unfulfilled vernalisation) | 100 |
| P5 | Thermal time from the onset of linear fill to maturity (8Cd) | 520 |
| G1 | Kernel number per unit stem + spike weight at anthesis (#/g) | 40 |
| G2 | Potential kernel growth rate (mg/(kernel d)) | 40 |
| G3 | Tiller death coefficient. Standard stem + spike weight when elongation ceases (g) | 2.1 |
| PHINT | Thermal time between the appearance of leaf tips (8Cd) | 95 |

Supplementary Material 2 Generic medium silty clay properties chosen as soil for calibration (θs – saturated soil water content).

| depth | clay |  | silt | θs |
| --- | --- | --- | --- | --- |
| [cm] | [ - ] |  | [ - ] | [ - ] |
| 5 | 0.23 |  | 0.39 | 0.46 |
| 15 | 0.23 |  | 0.39 | 0.46 |
| 30 | 0.23 |  | 0.39 | 0.46 |
| 45 | 0.25 |  | 0.41 | 0.46 |
| 60 | 0.25 |  | 0.41 | 0.46 |
| 90 | 0.31 |  | 0.45 | 0.46 |
| 120 | 0.21 |  | 0.34 | 0.46 |
| 150 | 0.26 |  | 0.37 | 0.46 |

Supplementary Material 3 Soil type used for validation and phenological modelling at DH, and UE model regions; derived from BUEK 1000n [26] (θs – saturated soil water content; θ_a_ available water content; ks- saturated permeability; CEC- cation exchange capacity).

| depth | horizon | | | clay | silt | | θs | | θa | | ks | | CEC | |
| --- | --- | --- | --- | --- | --- | --- | --- | --- | --- | --- | --- | --- | --- | --- |
| [cm] | |  | [ ] | | | [ ] | | [ ] | | [] | | [m/s] | | [cmol/kg] |
| 30 | | Ap | 0.22 | | | 0.49 | | 0.59 | | 36 | | 0.38 | | 10 |
| 40 | | Al | 0.11 | | | 0.28 | | 0.50 | | 32 | | 0.39 | | 5 |
| 80 | | Bv | 0.11 | | | 0.28 | | 0.50 | | 24 | | 0.2 | | 5 |
| 100 | | Bv | 0.07 | | | 0.26 | | 0.48 | | 24 | | 0.39 | | 3 |
| 200 | | C | 0.15 | | | 0.30 | | 0.48 | | 024 | | 0.25 | | 8 |

Supplementary Material 4 Soil type used for validation and phenological modelling at FL, and OS model region; derived from BUEK 1000n [26] (θs – saturated soil water content; θ_a_ available water content; ks- saturated permeability; CEC- cation exchange capacity).

| depth | horizon | clay | silt | θs | θa | ks | CEC |
| --- | --- | --- | --- | --- | --- | --- | --- |
| [cm] |  | [ ] | [ ] | [ ] | [ ] | [m/s] | [cmol/kg] |
| 30 | Ap | 0.12 | 0.26 | 0.48 | 24 | 2.55 | 4 |
| 40 | Al | 0.07 | 0.15 | 0.41 | 25 | 1.8 | 2 |
| 50 | Al | 0.07 | 0.15 | 0.41 | 24 | 1.8 | 2 |
| 60 | Bt | 0.11 | 0.23 | 0.43 | 18 | 1.32 | 2 |
| 80 | Bv | 0.07 | 0.15 | 0.41 | 18 | 1.8 | 8 |
| 200 | C | 0.15 | 0.28 | 0.43 | 18 | 0.26 | 7 |
