# Supplementary material for: Abundance of adverse environmental conditions during critical stages of crop production in Northern Germany
Source: Environ Sci Eur. 2018 Apr 2;30(1):10. doi: 10.1186/s12302-018-0138-0 (PMC5880854; doi:10.1186/s12302-018-0138-0)
Supplement: Supplementary file 2 — Additional file 2. Suppl Materials 5–12. [file 12302_2018_138_MOESM2_ESM.docx]

Additional file 2

Supplementary Material 5 Maize phenological trends identified for the baseline (BASE 1981- 2010) and the 3 projections (MAX, MED, MIN) of the projection period (2021-2050) for region DH..

|  |  | BASE | | | | | | MAX | | | | | | MED | | | | | | Min | | | | | |
| --- | --- | --- | --- | --- | --- | --- | --- | --- | --- | --- | --- | --- | --- | --- | --- | --- | --- | --- | --- | --- | --- | --- | --- | --- | --- |
| BBCH |  | Estimates | | | R² / df | p-value |  | Estimates | | | R² / df | p-value |  | Estimates | | | R² / df | p-value |  | Estimates | | | R² / df | p-value |  |
|  |  | [doy] [doy/y] | | | [] | [] |  | [doy] [doy/y] | | | [] | [] |  | [doy] [doy/y] | | | [] | [] |  | [doy] [doy/y] | | | [] | [] |  |
| 1 | Intercept | 124 | ± | 1 | 0.41 | 5.7E-39 | *** | 121 | ± | 3 | 0.45 | 2.7E-27 | *** | 119 | ± | 4 | 0.01 | 1.7E-23 | *** | 115 | ± | 4 | 0.00 | 3.9E-22 | *** |
|  | Slope | -0.27 | ± | 0.06 | 28 | 1.4E-04 | *** | -0.74 | ± | 0.16 | 28 | 5.1E-05 | *** | -0.08 | ± | 0.21 | 28 | 7.0E-01 |  | 0.05 | ± | 0.23 | 28 | 8.4E-01 |  |
| 11 | Intercept | 136 | ± | 2 | 0.39 | 1.9E-35 | *** | 131 | ± | 3 | 0.37 | 1.4E-28 | *** | 130 | ± | 4 | 0.01 | 3.7E-25 | *** | 128 | ± | 3 | 0.00 | 3.7E-25 | *** |
|  | Slope | -0.38 | ± | 0.09 | 28 | 2.3E-04 | *** | -0.61 | ± | 0.15 | 28 | 3.8E-04 | *** | -0.08 | ± | 0.20 | 28 | 7.0E-01 |  | -0.03 | ± | 0.20 | 28 | 8.6E-01 |  |
| 31 | Intercept | 163 | ± | 2 | 0.07 | 1.9E-33 | *** | 160 | ± | 2 | 0.46 | 1.4E-33 | *** | 160 | ± | 3 | 0.03 | 1.1E-28 | *** | 157 | ± | 3 | 0.00 | 9.2E-29 | *** |
|  | Slope | -0.18 | ± | 0.13 | 28 | 1.7E-01 |  | -0.60 | ± | 0.12 | 28 | 3.5E-05 | *** | -0.17 | ± | 0.18 | 28 | 3.6E-01 |  | -0.06 | ± | 0.18 | 28 | 7.4E-01 |  |
| 61 | Intercept | 210 | ± | 2 | 0.20 | 1.6E-36 | *** | 207 | ± | 2 | 0.51 | 3.0E-35 | *** | 206 | ± | 3 | 0.07 | 1.5E-31 | *** | 201 | ± | 3 | 0.00 | 7.0E-32 | *** |
|  | Slope | -0.33 | ± | 0.13 | 28 | 1.4E-02 | * | -0.75 | ± | 0.14 | 28 | 8.9E-06 | *** | -0.26 | ± | 0.19 | 28 | 1.7E-01 |  | -0.07 | ± | 0.18 | 28 | 7.1E-01 |  |
| 70 | Intercept | 223 | ± | 2 | 0.20 | 1.6E-36 | *** | 221 | ± | 3 | 0.53 | 2.8E-35 | *** | 218 | ± | 3 | 0.08 | 2.6E-32 | *** | 213 | ± | 3 | 0.00 | 7.3E-32 | *** |
|  | Slope | -0.36 | ± | 0.13 | 28 | 1.3E-02 | * | -0.82 | ± | 0.15 | 28 | 5.9E-06 | *** | -0.28 | ± | 0.19 | 28 | 1.4E-01 |  | -0.01 | ± | 0.19 | 28 | 9.5E-01 |  |
| 99 | Intercept | 288 | ± | 6 | 0.17 | 3.4E-28 | *** | 280 | ± | 5 | 0.54 | 3.3E-31 | *** | 272 | ± | 5 | 0.17 | 1.6E-29 | *** | 263 | ± | 6 | 0.00 | 1.3E-26 | *** |

Supplementary Material 6 Maize phenological trends identified for the baseline (BASE 1981- 2010) and the 3 projections (MAX, MED, MIN) of the scenario period (2021-2050) for model region Uelzen (UE).

|  |  | BASE | | | | | | | | | MIN | | | | | | | | | | | | | | MED | | | | | | | | | | | | | | | | MAX | | | | | | | | | | | | | | | |  |  |
| --- | --- | --- | --- | --- | --- | --- | --- | --- | --- | --- | --- | --- | --- | --- | --- | --- | --- | --- | --- | --- | --- | --- | --- | --- | --- | --- | --- | --- | --- | --- | --- | --- | --- | --- | --- | --- | --- | --- | --- | --- | --- | --- | --- | --- | --- | --- | --- | --- | --- | --- | --- | --- | --- | --- | --- | --- | --- | --- |
| BBCH |  | Estimates | | R² / df | | | p-value | |  | | Estimates | | | | | R² / df | | | p-value | | | |  | | Estimates | | | | | | | | R² / df | | p-value | | |  | | | Estimates | | | | | | | R² / df | | | P-value | | |  | | |  |  |
|  |  | [doy] [doy/y] | | [] | | | [] | |  | | [doy] [doy/y] | | | | | [] | | | [] | | | |  | | [doy] [doy/y] | | | | | | | | [] | | [] | | |  | | | [doy] [doy/y] | | | | | | | [] | | | [] | | |  | | |  |  |
| 1 | Intercept | 124 | ± | | 1 | 0.20 | | 1.7E-36 | | *** | | 124 | ± | 3 | | | 0.49 | | | 8.9E-28 | | *** | | | | 122 | | ± | | 4 | | 0.03 | | | | 3.7E-23 | | | *** | | | 119 | | ± | | 4 | | | 0.00 | | | 4.0E-24 | | | *** | | |  |
|  | Slope | -0.20 | ± | | 0.07 | 28 | | 1.4E-02 | | * | | -0.79 | ± | | 0.15 | | | 28 | | | 1.9E-05 | | | *** | | | -0.21 | | ± | | 0.22 | | | 28 | | | 3.6E-01 | | |  | | | -0.04 | | ± | | 0.20 | | | 28 | | | 8.5E-01 | | |  | | |
| 11 | Intercept | 136 | ± | | 2 | 0.21 | | 6.7E-35 | | *** | | 134 | ± | | 3 | | | 0.34 | | | 3.3E-28 | | | *** | | | 135 | | ± | | 4 | | | 0.05 | | | 1.4E-24 | | | *** | | | 130 | | ± | | 3 | | | 0.00 | | | 1.1E-26 | | | *** | | |
|  | Slope | -0.26 | ± | | 0.09 | 28 | | 1.1E-02 | | * | | -0.61 | ± | | 0.16 | | | 28 | | | 7.3E-04 | | | *** | | | -0.26 | | ± | | 0.22 | | | 28 | | | 2.5E-01 | | |  | | | -0.05 | | ± | | 0.18 | | | 28 | | | 7.8E-01 | | |  | | |
| 31 | Intercept | 166 | ± | | 2 | 0.07 | | 4.4E-34 | | *** | | 163 | ± | | 2 | | | 0.51 | | | 4.1E-34 | | | *** | | | 165 | | ± | | 4 | | | 0.08 | | | 1.9E-27 | | | *** | | | 161 | | ± | | 3 | | | 0.01 | | | 1.0E-29 | | | *** | | |
|  | Slope | -0.17 | ± | | 0.12 | 28 | | 1.7E-01 | |  | | -0.64 | ± | | 0.12 | | | 28 | | | 1.1E-05 | | | *** | | | -0.34 | | ± | | 0.21 | | | 28 | | | 1.2E-01 | | |  | | | -0.08 | | ± | | 0.17 | | | 28 | | | 6.3E-01 | | |  | | |
| 61 | Intercept | 214 | ± | | 2 | 0.14 | | 5.4E-36 | | *** | | 210 | ± | | 2 | | | 0.57 | | | 1.0E-36 | | | *** | | | 212 | | ± | | 4 | | | 0.09 | | | 1.7E-30 | | | *** | | | 207 | | ± | | 3 | | | 0.03 | | | 2.8E-32 | | | *** | | |
|  | Slope | -0.29 | ± | | 0.13 | 28 | | 4.1E-02 | | * | | -0.75 | ± | | 0.12 | | | 28 | | | 1.6E-06 | | | *** | | | -0.36 | | ± | | 0.21 | | | 28 | | | 9.9E-02 | | | . | | | -0.17 | | ± | | 0.18 | | | 28 | | | 3.6E-01 | | |  | | |
| 70 | Intercept | 227 | ± | | 3 | 0.15 | | 2.5E-35 | | *** | | 223 | ± | | 2 | | | 0.58 | | | 7.5E-37 | | | *** | | | 225 | | ± | | 4 | | | 0.11 | | | 3.6E-31 | | | *** | | | 219 | | ± | | 3 | | | 0.00 | | | 9.8E-32 | | | *** | | |
|  | Slope | -0.33 | ± | | 0.15 | 28 | | 3.7E-02 | | * | | -0.81 | ± | | 0.13 | | | 28 | | | 1.0E-06 | | | *** | | | -0.39 | | ± | | 0.21 | | | 28 | | | 7.3E-02 | | | . | | | -0.07 | | ± | | 0.20 | | | 28 | | | 7.2E-01 | | |  | | |
| 99 | Intercept | 298 | ± | | 7 | 0.12 | | 9.1E-28 | | *** | | 288 | ± | | 5 | | | 0.53 | | | 5.4E-30 | | | *** | | | 287 | | ± | | 6 | | | 0.20 | | | 6.5E-28 | | | *** | | | 278 | | ± | | 8 | | | 0.00 | | | 1.6E-24 | | | *** | | |
|  | Slope | -0.73 | ± | | 0.37 | 28 | | 5.7E-02 | | . | | -1.67 | ± | | 0.30 | | | 28 | | | 5.1E-06 | | | *** | | | -0.92 | | ± | | 0.35 | | | 28 | | | 1.4E-02 | | | * | | | 0.00 | | ± | | 0.45 | | | 28 | | | 1.0E+00 | | |  | | |

Supplementary Material 7 Maize phenological trends identified for the baseline (BASE 1981- 2010) and the 3 projections (MAX, MED, MIN) of the scenario period (2021-2050) for model region Fläming (FL).

|  |  | | BASE | | | | | | | | | | | | | | | | | | | MIN | | | | | | | | | | | | | | | | | | | | | MED | | | | | | | | | | | | | | | | | | | | | | MAX | | | | | | | | | | | | | | | | | | | | |
| --- | --- | --- | --- | --- | --- | --- | --- | --- | --- | --- | --- | --- | --- | --- | --- | --- | --- | --- | --- | --- | --- | --- | --- | --- | --- | --- | --- | --- | --- | --- | --- | --- | --- | --- | --- | --- | --- | --- | --- | --- | --- | --- | --- | --- | --- | --- | --- | --- | --- | --- | --- | --- | --- | --- | --- | --- | --- | --- | --- | --- | --- | --- | --- | --- | --- | --- | --- | --- | --- | --- | --- | --- | --- | --- | --- | --- | --- | --- | --- | --- | --- | --- | --- | --- | --- |
| BBCH |  | | Estimates | | | | | | | | | R² / df | | | | p-value | | | |  | | Estimates | | | | | | | | | | | R² / df | | | | p-value | | |  | | | Estimates | | | | | | | | | | R² / df | | | | p-value | | | |  | | | | Estimates | | | | | | | | | R² / df | | | | P-value | | | |  | | | |
|  |  | | [doy] [doy/y] | | | | | | | | | [] | | | | [] | | | |  | | [doy] [doy/y] | | | | | | | | | | | [] | | | | [] | | |  | | | [doy] [doy/y] | | | | | | | | | | [] | | | | [] | | | |  | | | | [doy] [doy/y] | | | | | | | | | [] | | | | [] | | | |  | | | |
| 1 | Intercept | | 126 | | ± | | 1 | | 0.51 | | | | 1.2E-35 | | | | *** | | | | 117 | | | ± | | | 2 | | | 0.52 | | | | 4.7E-30 | | | | *** | | | 116 | | | ± | | | 3 | | | 0.08 | | | | 1.2E-24 | | | | *** | | | | 117 | | | | ± | | | 4 | | | 0.03 | | | | 3.1E-23 | | | | *** | | | |  |  |
|  | Slope | | -0.44 | | ± | | 0.08 | | | 28 | | | | 1.0E-05 | | | | *** | | | | -0.66 | | | ± | | | 0.12 | | | 28 | | | | 6.1E-06 | | | | *** | | | -0.29 | | | ± | | | 0.19 | | | 28 | | | | 1.3E-01 | | | |  | | | | -0.21 | | | | ± | | | 0.21 | | | 28 | | | | 3.3E-01 | | | |  | | | |  |
| 11 | Intercept | | 136 | | ± | | 2 | | | 0.30 | | | | 4.7E-33 | | | | *** | | | | 127 | | | ± | | | 2 | | | 0.43 | | | | 5.9E-30 | | | | *** | | | 126 | | | ± | | | 3 | | | 0.04 | | | | 4.3E-27 | | | | *** | | | | 130 | | | | ± | | | 3 | | | 0.10 | | | | 1.9E-26 | | | | *** | | | |  |
|  | Slope | | -0.37 | | ± | | 0.11 | | | 28 | | | | 1.9E-03 | | | | ** | | | | -0.61 | | | ± | | | 0.13 | | | 28 | | | | 7.8E-05 | | | | *** | | | -0.18 | | | ± | | | 0.16 | | | 28 | | | | 2.8E-01 | | | |  | | | | -0.31 | | | | ± | | | 0.18 | | | 28 | | | | 9.2E-02 | | | | . | | | |  |
| 31 | | Intercept | | 161 | | ± | | 3 | | | 0.06 | | | | 6.9E-32 | | | | *** | | | | 154 | | | ± | | | 2 | | | 0.41 | | | | 4.7E-33 | | | | *** | | | 155 | | | ± | | | 3 | | | 0.08 | | | | 1.3E-29 | | | | *** | | | | 155 | | | | ± | | | 3 | | | | 0.04 | | | | 6.0E-29 | | | | *** | | |
|  | | Slope | | -0.18 | | ± | | 0.14 | | | 28 | | | | 2.1E-01 | | | |  | | | | -0.55 | | | ± | | | 0.12 | | | 28 | | | | 1.3E-04 | | | | *** | | | -0.26 | | | ± | | | 0.16 | | | 28 | | | | 1.3E-01 | | | |  | | | | -0.19 | | | | ± | | | 0.17 | | | | 28 | | | | 3.0E-01 | | | |  | | |
| 61 | | Intercept | | 206 | | ± | | 3 | | | 0.15 | | | | 7.0E-35 | | | | *** | | | | 197 | | | ± | | | 2 | | | 0.51 | | | | 7.3E-37 | | | | *** | | | 197 | | | ± | | | 3 | | | 0.07 | | | | 5.1E-32 | | | | *** | | | | 197 | | | | ± | | | 3 | | | | 0.02 | | | | 1.6E-30 | | | | *** | | |
|  | | Slope | | -0.32 | | ± | | 0.14 | | | 28 | | | | 3.2E-02 | | | | * | | | | -0.62 | | | ± | | | 0.12 | | | 28 | | | | 1.1E-05 | | | | *** | | | -0.24 | | | ± | | | 0.17 | | | 28 | | | | 1.7E-01 | | | |  | | | | -0.14 | | | | ± | | | 0.19 | | | | 28 | | | | 4.8E-01 | | | |  | | |
| 70 | | Intercept | | 219 | | ± | | 3 | | | 0.17 | | | | 6.2E-35 | | | | *** | | | | 209 | | | ± | | | 2 | | | 0.53 | | | | 5.6E-37 | | | | *** | | | 210 | | | ± | | | 3 | | | 0.09 | | | | 8.6E-33 | | | | *** | | | | 208 | | | | ± | | | 4 | | | | 0.01 | | | | 9.2E-31 | | | | *** | | |
|  | | Slope | | -0.35 | | ± | | 0.15 | | | 28 | | | | 2.6E-02 | | | | * | | | | -0.68 | | | ± | | | 0.12 | | | 28 | | | | 4.8E-06 | | | | *** | | | -0.28 | | | ± | | | 0.17 | | | 28 | | | | 1.1E-01 | | | |  | | | | -0.11 | | | | ± | | | 0.20 | | | | 28 | | | | 6.0E-01 | | | |  | | |
| 99 | | Intercept | | 276 | | ± | | 5 | | | 0.19 | | | | 2.4E-29 | | | | *** | | | | 253 | | | ± | | | 3 | | | 0.59 | | | | 5.8E-37 | | | | *** | | | 258 | | | ± | | | 5 | | | 0.17 | | | | 5.3E-30 | | | | *** | | | | 253 | | | | ± | | | 5 | | | | 0.00 | | | | 2.9E-28 | | | | *** | | |
|  | | Slope | | -0.77 | | ± | | 0.30 | | | 28 | | | | 1.6E-02 | | | | * | | | | -0.94 | | | ± | | | 0.15 | | | 28 | | | | 6.5E-07 | | | | *** | | | -0.64 | | | ± | | | 0.27 | | | 28 | | | | 2.3E-02 | | | | * | | | | -0.05 | | | | ± | | | 0.30 | | | | 28 | | | | 8.7E-01 | | | |  | | |

Supplementary Material 8 Maize phenological trends identified for the baseline (BASE 1981- 2010) and the 3 projections (MAX, MED, MIN) of the scenario period (2021-2050) for model region Oder-Spree (OS).

|  |  | BASE | | | | | | MIN | | | | | | MED | | | | | | MAX | | | | | |
| --- | --- | --- | --- | --- | --- | --- | --- | --- | --- | --- | --- | --- | --- | --- | --- | --- | --- | --- | --- | --- | --- | --- | --- | --- | --- |
| BBCH |  | Estimates | | | R² / df | p-value |  | Estimates | | | R² / df | p-value |  | Estimates | | | R² / df | p-value |  | Estimates | | | R² / df | P-value |  |
|  |  | [doy] [doy/y] | | | [] | [] |  | [doy] [doy/y] | | | [] | [] |  | [doy] [doy/y] | | | [] | [] |  | [doy] [doy/y] | | | [] | [] |  |
| 1 | Intercept | 124 | ± | 2 | 0,11 | 1.1E-31 | *** | 121 | ± | 2 | 0.62 | 2.4E-30 | *** | 121 | ± | 4 | 0.12 | 2.7E-23 | *** | 115 | ± | 4 | 0.01 | 2.0E-23 | *** |
|  | Slope | -0.21 | ± | 0.11 | 28 | 6.9E-02 | . | -0.81 | ± | 0.12 | 28 | 2.7E-07 | *** | -0.43 | ± | 0.22 | 28 | 5.8E-02 | . | -0.11 | ± | 0.21 | 28 | 6.1E-01 |  |
| 11 | Intercept | 136 | ± | 2 | 0.26 | 2.3E-33 | *** | 130 | ± | 2 | 0.53 | 7.6E-31 | *** | 133 | ± | 4 | 0.11 | 3.6E-24 | *** | 127 | ± | 3 | 0.03 | 7.5E-28 | *** |
|  | Slope | -0.33 | ± | 0.11 | 28 | 4.2E-03 | ** | -0.70 | ± | 0.13 | 28 | 5.3E-06 | *** | -0.41 | ± | 0.22 | 28 | 7.6E-02 | . | -0.15 | ± | 0.16 | 28 | 3.6E-01 |  |
| 31 | Intercept | 162 | ± | 2 | 0.04 | 1.7E-32 | *** | 158 | ± | 2 | 0.56 | 1.4E-34 | *** | 160 | ± | 4 | 0.14 | 6.3E-27 | *** | 156 | ± | 3 | 0.05 | 6.6E-31 | *** |
|  | Slope | -0.16 | ± | 0.14 | 28 | 2.6E-01 |  | -0.66 | ± | 0.11 | 28 | 2.0E-06 | *** | -0.45 | ± | 0.21 | 28 | 4.4E-02 | * | -0.18 | ± | 0.15 | 28 | 2.5E-01 |  |
| 61 | Intercept | 206 | ± | 3 | 0.07 | 1.2E-33 | *** | 199 | ± | 2 | 0.61 | 4.5E-38 | *** | 201 | ± | 4 | 0.12 | 4.0E-30 | *** | 198 | ± | 3 | 0.06 | 9.2E-34 | *** |
|  | Slope | -0.23 | ± | 0.16 | 28 | 1.5E-01 |  | -0.70 | ± | 0.11 | 28 | 3.1E-07 | *** | -0.39 | ± | 0.21 | 28 | 6.6E-02 | . | -0.20 | ± | 0.15 | 28 | 1.9E-01 |  |
| 70 | Intercept | 219 | ± | 3 | 0.11 | 1.2E-34 | *** | 211 | ± | 2 | 0.59 | 1.4E-37 | *** | 213 | ± | 4 | 0.14 | 4.2E-31 | *** | 210 | ± | 3 | 0.06 | 4.4E-34 | *** |
|  | Slope | -0.29 | ± | 0.15 | 28 | 6.7E-02 | . | -0.74 | ± | 0.12 | 28 | 6.1E-07 | *** | -0.43 | ± | 0.20 | 28 | 4.0E-02 | * | -0.20 | ± | 0.15 | 28 | 2.1E-01 |  |
| 99 | Intercept | 277 | ± | 7 | 0.09 | 9.7E-27 | *** | 257 | ± | 2 | 0.73 | 5.7E-39 | *** | 263 | ± | 5 | 0.22 | 2.5E-29 | *** | 254 | ± | 5 | 0.01 | 4.4E-30 | *** |
|  | Slope | -0.64 | ± | 0.37 | 28 | 1.0E-01 |  | -1.09 | ± | 0.13 | 28 | 2.3E-09 | *** | -0.81 | ± | 0.29 | 28 | 8.5E-03 | ** | -0.13 | ± | 0.26 | 28 | 6.2E-01 |  |

Supplementary Material 9 Wheat phenological trends identified for the baseline (BASE 1981- 2010) and the 3 projections (MAX, MED, MIN) of the projection period (2021-2050) for model DH.

|  |  |  |  |  |  |  |  |  |  |  |  |  |  |  |  |  |  |  |  |  |  |  |  |  |  |
| --- | --- | --- | --- | --- | --- | --- | --- | --- | --- | --- | --- | --- | --- | --- | --- | --- | --- | --- | --- | --- | --- | --- | --- | --- | --- |
|  |  | BASE | | | | | | MIN | | | | | | MED | | | | | | MAX | | | | | |
| BBCH |  | Estimate | | | R² / df | p-value |  | Estimate | | | R² / df | p-value |  | Estimate | | | R² / df | p-value |  | Estimate | | | R² / df | p-value |  |
|  |  | [doy] [doy/y] | | | [] | [] |  | [doy] [doy/y] | | | [] | [] |  | [doy] [doy/y] | | | [] | [] |  | [doy] [doy/y] | | | [] | [] |  |
| 31 | Intercept | 122 | ± | 40 | 0.21 | 4.7E-03 | ** | 120 | ± | 26 | 0.38 | 8.1E-05 | *** | 118 | ± | 2 | 0.05 | 2.6E-30 | *** | 117 | ± | 2 | 0.01 | 3.7E-29 | *** |
|  | Slope | -0.90 | ± | 0.33 | 27 | 1.2E-02 | * | -0.92 | ± | 0.23 | 27 | 3.8E-04 | *** | -0.14 | ± | 0.11 | 27 | 2.4E-01 |  | -0.08 | ± | 0.13 | 27 | 5.3E-01 |  |
| 51 | Intercept | 86 | ± | 40 | 0.10 | 4.1E-02 | * | 161 | ± | 31 | 0.45 | 2.1E-05 | *** | 152 | ± | 2 | 0.05 | 2.8E-31 | *** | 151 | ± | 2 | 0.01 | 4.2E-31 | *** |
|  | Slope | -0.47 | ± | 0.26 | 27 | 8.8E-02 | . | -1.00 | ± | 0.21 | 27 | 7.4E-05 | *** | -0.17 | ± | 0.14 | 27 | 2.2E-01 |  | -0.06 | ± | 0.14 | 27 | 6.5E-01 |  |
| 61 | Intercept | 100 | ± | 39 | 0.15 | 1.6E-02 | * | 187 | ± | 36 | 0.45 | 2.1E-05 | *** | 170 | ± | 2 | 0.09 | 1.0E-32 | *** | 167 | ± | 2 | 0.01 | 3.8E-32 | *** |
|  | Slope | -0.50 | ± | 0.23 | 27 | 3.7E-02 | * | -1.05 | ± | 0.22 | 27 | 6.3E-05 | *** | -0.22 | ± | 0.13 | 27 | 1.1E-01 |  | -0.07 | ± | 0.14 | 27 | 6.3E-01 |  |
| 74 | Intercept | 118 | ± | 43 | 0.18 | 1.0E-02 | * | 203 | ± | 35 | 0.52 | 3.0E-06 | *** | 191 | ± | 2 | 0.08 | 1.9E-33 | *** | 189 | ± | 2 | 0.01 | 4.0E-33 | *** |
|  | Slope | -0.54 | ± | 0.23 | 27 | 2.3E-02 | * | -1.02 | ± | 0.19 | 27 | 9.5E-06 | *** | -0.21 | ± | 0.14 | 27 | 1.5E-01 |  | -0.07 | ± | 0.14 | 27 | 6.5E-01 |  |
| 99 | Intercept | 222 | ± | 2 | 0.22 | 3.0E-35 | *** | 203 | ± | 33 | 0.54 | 1.8E-06 | *** | 217 | ± | 3 | 0.08 | 1.5E-34 | *** | 214 | ± | 3 | 0.00 | 1.5E-33 | *** |
|  | Slope | -0.39 | ± | 0.14 | 27 | 1.0E-02 | * | -0.88 | ± | 0.16 | 27 | 5.9E-06 | *** | -0.23 | ± | 0.15 | 27 | 1.3E-01 |  | 0.03 | ± | 0.16 | 27 | 8.3E-01 |  |

Supplementary Material 10 Wheat phenological trends identified for the baseline (BASE 1981- 2010) and the 3 projections (MAX, MED, MIN) of the scenario period (2021-2050) for model region Uelzen (UE).

|  |  | BASE | | | | | | | | | MAX | | | | | | | | | | MED | | | | | | | Min | | | | | | | | | |
| --- | --- | --- | --- | --- | --- | --- | --- | --- | --- | --- | --- | --- | --- | --- | --- | --- | --- | --- | --- | --- | --- | --- | --- | --- | --- | --- | --- | --- | --- | --- | --- | --- | --- | --- | --- | --- | --- |
| BBCH |  | Estimate | | | R² / df | | p-value | |  | | Estimate | | | | R² / df | | p-value | |  | | Estimate | | | | R² / df | p-value |  | Estimate | | | | R² / df | | P-value | |  | |
|  |  | [doy] [doy/y] | | | [] | | [] | |  | | [doy] [doy/y] | | | | [] | | [] | |  | | [doy] [doy/y] | | | | [] | [] |  | [doy] [doy/y] | | | | [] | | [] | |  | |
| 31 | Intercept | 125 | ± | 2 | | 0.26 | | 8.8E-34 | | *** | | 124 | ± | 27 | | 0.37 | | 1.1E-04 | | *** | | 120 | ± | 2 | 0.03 | 3.6E-30 | *** | 120 | ± | 2 | 0.03 | | 1.8E-29 | | *** | |  |
|  | Slope | -0.28 | ± | 0.09 | | 27 | | 4.8E-03 | | ** | | -0.95 | ± | 0.24 | | 27 | | 4.7E-04 | | *** | | -0.11 | ± | 0.12 | 27 | 3.6E-01 |  | -0.12 | ± | 0.13 | 27 | | 3.4E-01 | |  | |  |
| 51 | Intercept | 160 | ± | 2 | | 0.19 | | 2.5E-34 | | *** | | 162 | ± | 31 | | 0.46 | | 1.5E-05 | | *** | | 155 | ± | 2 | 0.05 | 1.2E-30 | *** | 154 | ± | 2 | 0.01 | | 1.1E-30 | | *** | |  |
|  | Slope | -0.27 | ± | 0.11 | | 27 | | 1.9E-02 | | * | | -1.00 | ± | 0.21 | | 27 | | 5.4E-05 | | *** | | -0.17 | ± | 0.15 | 27 | 2.4E-01 |  | -0.09 | ± | 0.14 | 27 | | 5.3E-01 | |  | |  |
| 61 | Intercept | 179 | ± | 2 | | 0.22 | | 1.2E-34 | | *** | | 182 | ± | 34 | | 0.48 | | 1.1E-05 | | *** | | 173 | ± | 2 | 0.07 | 5.9E-32 | *** | 172 | ± | 2 | 0.03 | | 2.8E-32 | | *** | |  |
|  | Slope | -0.33 | ± | 0.12 | | 27 | | 1.0E-02 | | * | | -1.01 | ± | 0.20 | | 27 | | 3.4E-05 | | *** | | -0.21 | ± | 0.14 | 27 | 1.6E-01 |  | -0.13 | ± | 0.14 | 27 | | 3.8E-01 | |  | |  |
| 74 | Intercept | 195 | ± | 2 | | 0.25 | | 3.3E-36 | | *** | | 205 | ± | 34 | | 0.53 | | 2.3E-06 | | *** | | 194 | ± | 3 | 0.05 | 3.4E-33 | *** | 193 | ± | 3 | 0.02 | | 9.8E-33 | | *** | |  |
|  | Slope | -0.34 | ± | 0.11 | | 27 | | 5.3E-03 | | ** | | -1.05 | ± | 0.19 | | 27 | | 7.2E-06 | | *** | | -0.18 | ± | 0.15 | 27 | 2.2E-01 |  | -0.10 | ± | 0.15 | 27 | | 5.0E-01 | |  | |  |
| 99 | Intercept | 233 | ± | 2 | | 0.30 | | 2.7E-37 | | *** | | 211 | ± | 34 | | 0.55 | | 1.5E-06 | | *** | | 221 | ± | 3 | 0.06 | 1.8E-34 | *** | 219 | ± | 3 | 0.00 | | 1.0E-33 | | *** | |  |
|  | Slope | -0.42 | ± | 0.12 | | 27 | | 2.1E-03 | | ** | | -0.91 | ± | 0.16 | | 27 | | 4.6E-06 | | *** | | -0.20 | ± | 0.15 | 27 | 2.0E-01 |  | -0.02 | ± | 0.16 | 27 | | 9.0E-01 | |  | |  |

Supplementary Material 11 Wheat phenological trends identified for the baseline (BASE 1981- 2010) and the 3 projections (MAX, MED, MIN) of the scenario period (2021-2050) for model region Fläming (FL).

|  |  | BASE | | | | | | | | MAX | | | | | | | MED | | | | | | | | | | Min | | | | | | | | | |
| --- | --- | --- | --- | --- | --- | --- | --- | --- | --- | --- | --- | --- | --- | --- | --- | --- | --- | --- | --- | --- | --- | --- | --- | --- | --- | --- | --- | --- | --- | --- | --- | --- | --- | --- | --- | --- |
| BBCH |  | Estimate | | | R² / df | | p-value | |  | Estimate | | | R² / df | p-value |  | | Estimate | | | | R² / df | | p-value | |  | | Estimate | | | | R² / df | | P-value | |  | |
|  |  | [doy] [doy/y] | | | [] | | [] | |  | [doy] [doy/y] | | | [] | [] |  | [doy] [doy/y] | | | | [] | | [] | |  | | [doy] [doy/y] | | | | [] | | [] | |  | |  |
| 31 | Intercept | 129 | ± | 2 | | 0.28 | | 4.8E-34 | *** | 123 | ± | 2 | 0.42 | 1.1E-32 | *** | | 124 | ± | 2 | | 0.07 | | 8.3E-31 | | *** | | 123 | ± | 2 | | 0.02 | | 1.8E-30 | | *** | |
|  | Slope | -0.29 | ± | 0.09 | | 27 | | 3.1E-03 | ** | -0.43 | ± | 0.10 | 27 | 1.6E-04 | *** | | -0.16 | ± | 0.11 | | 27 | | 1.7E-01 | |  | | -0.09 | ± | 0.12 | | 27 | | 4.4E-01 | |  | |
| 51 | Intercept | 156 | ± | 2 | | 0.10 | | 2.2E-32 | *** | 150 | ± | 2 | 0.47 | 3.1E-35 | *** | | 151 | ± | 2 | | 0.07 | | 1.5E-31 | | *** | | 150 | ± | 2 | | 0.02 | | 2.0E-31 | | *** | |
|  | Slope | -0.22 | ± | 0.13 | | 27 | | 8.8E-02 | . | -0.47 | ± | 0.10 | 27 | 3.6E-05 | *** | | -0.19 | ± | 0.13 | | 27 | | 1.6E-01 | |  | | -0.10 | ± | 0.13 | | 27 | | 4.6E-01 | |  | |
| 61 | Intercept | 174 | ± | 2 | | 0.15 | | 1.0E-32 | *** | 167 | ± | 2 | 0.49 | 2.4E-36 | *** | | 168 | ± | 2 | | 0.09 | | 3.1E-32 | | *** | | 166 | ± | 2 | | 0.01 | | 1.0E-32 | | *** | |
|  | Slope | -0.30 | ± | 0.14 | | 27 | | 3.7E-02 | * | -0.49 | ± | 0.10 | 27 | 2.4E-05 | *** | | -0.22 | ± | 0.14 | | 27 | | 1.2E-01 | |  | | -0.08 | ± | 0.13 | | 27 | | 5.6E-01 | |  | |
| 74 | Intercept | 195 | ± | 2 | | 0.18 | | 3.6E-34 | *** | 187 | ± | 1 | 0.60 | 2.8E-39 | *** | | 188 | ± | 2 | | 0.08 | | 3.1E-33 | | *** | | 187 | ± | 2 | | 0.02 | | 1.3E-33 | | *** | |
|  | Slope | -0.33 | ± | 0.14 | | 27 | | 2.3E-02 | * | -0.54 | ± | 0.08 | 27 | 6.9E-07 | *** | | -0.22 | ± | 0.14 | | 27 | | 1.4E-01 | |  | | -0.11 | ± | 0.14 | | 27 | | 4.3E-01 | |  | |
| 99 | Intercept | 222 | ± | 2 | | 0.22 | | 3.0E-35 | *** | 213 | ± | 2 | 0.61 | 1.1E-39 | *** | | 214 | ± | 2 | | 0.11 | | 1.3E-34 | | *** | | 211 | ± | 2 | | 0.00 | | 1.3E-34 | | *** | |
|  | Slope | -0.39 | ± | 0.14 | | 27 | | 1.0E-02 | * | -0.60 | ± | 0.09 | 27 | 5.7E-07 | *** | | -0.26 | ± | 0.14 | | 27 | | 8.2E-02 | | . | | -0.01 | ± | 0.14 | | 27 | | 9.6E-01 | |  | |

Supplementary Material 12 Wheat phenological trends identified for the baseline (BASE 1981- 2010) and the 3 projections (MAX, MED, MIN) of the scenario period (2021-2050) for model region Oder-Spree (OS).

|  |  | BASE | | | | | | | | MAX | | | | | | | | | | MED | | | | | | Min | | | | | |
| --- | --- | --- | --- | --- | --- | --- | --- | --- | --- | --- | --- | --- | --- | --- | --- | --- | --- | --- | --- | --- | --- | --- | --- | --- | --- | --- | --- | --- | --- | --- | --- |
| BBCH |  | Estimate | | | R² / n | | p-value | |  | Estimate | | | | R² / n | | p-value | |  | | Estimate | | | R² / n | p-value |  | Estimate | | | R² / n | p-value |  |
|  |  | [doy] [doy/y] | | | [] | | [] | |  | [doy] [doy/y] | | | | [] | | [] | |  | | [doy] [doy/y] | | | [] | [] |  | [doy] [doy/y] | | | [] | [] |  |
| 31 | Intercept | 123 | ± | 1 | | 0.21 | | 6.0E-34 | *** | 136 | ± | 23 | 0.50 | | 3.2E-06 | | *** | | 123 | | ± | 2 | 0.07 | 4.6E-31 | *** | 118 | ± | 2 | 0.01 | 1.1E-29 | *** |
|  | Slope | -0.24 | ± | 0.09 | | 27 | | 1.1E-02 | * | -1.08 | ± | 0.21 | 27 | | 1.8E-05 | | *** | | -0.16 | | ± | 0.11 | 27 | 1.7E-01 |  | -0.05 | ± | 0.12 | 27 | 6.7E-01 |  |
| 51 | Intercept | 156 | ± | 2 | | 0.09 | | 8.3E-33 | *** | 174 | ± | 28 | 0.54 | | 1.5E-06 | | *** | | 152 | | ± | 2 | 0.09 | 2.3E-31 | *** | 150 | ± | 2 | 0.03 | 7.3E-32 | *** |
|  | Slope | -0.20 | ± | 0.12 | | 27 | | 1.1E-01 |  | -1.10 | ± | 0.20 | 27 | | 6.1E-06 | | *** | | -0.22 | | ± | 0.13 | 27 | 1.1E-01 |  | -0.11 | ± | 0.13 | 27 | 4.1E-01 |  |
| 61 | Intercept | 174 | ± | 2 | | 0.18 | | 7.1E-34 | *** | 189 | ± | 31 | 0.53 | | 2.1E-06 | | *** | | 168 | | ± | 2 | 0.09 | 4.6E-32 | *** | 166 | ± | 2 | 0.01 | 2.9E-33 | *** |
|  | Slope | -0.31 | ± | 0.12 | | 27 | | 2.0E-02 | * | -1.09 | ± | 0.20 | 27 | | 7.3E-06 | | *** | | -0.23 | | ± | 0.14 | 27 | 1.2E-01 |  | -0.08 | ± | 0.12 | 27 | 5.5E-01 |  |
| 74 | Intercept | 195 | ± | 2 | | 0.22 | | 6.0E-35 | *** | 231 | ± | 30 | 0.66 | | 2.5E-08 | | *** | | 188 | | ± | 2 | 0.08 | 3.5E-33 | *** | 187 | ± | 2 | 0.03 | 1.0E-33 | *** |
|  | Slope | -0.35 | ± | 0.13 | | 27 | | 1.1E-02 | * | -1.21 | ± | 0.17 | 27 | | 8.6E-08 | | *** | | -0.22 | | ± | 0.14 | 27 | 1.3E-01 |  | -0.12 | ± | 0.14 | 27 | 3.6E-01 |  |
| 99 | Intercept | 223 | ± | 2 | | 0.26 | | 1.1E-35 | *** | 243 | ± | 31 | 0.67 | | 2.2E-08 | | *** | | 214 | | ± | 2 | 0.10 | 7.0E-35 | *** | 212 | ± | 2 | 0.00 | 1.2E-34 | *** |
|  | Slope | -0.42 | ± | 0.14 | | 27 | | 4.3E-03 | ** | -1.11 | ± | 0.15 | 27 | | 7.0E-08 | | *** | | -0.24 | | ± | 0.14 | 27 | 9.4E-02 | . | -0.04 | ± | 0.14 | 27 | 7.7E-01 |  |
